# Use of Naturally Available Reference Targets to Calibrate Airborne Laser Scanning Intensity Data

**DOI:** 10.3390/s90402780

**Published:** 2009-04-20

**Authors:** Ants Vain, Sanna Kaasalainen, Ulla Pyysalo, Anssi Krooks, Paula Litkey

**Affiliations:** Department of Remote Sensing and Photogrammetry, Finnish Geodetic Institute, Geodeetinrinne 2, 02431 Masala, Finland. E-Mails: sanna.kaasalainen@fgi.fi; ulla.pyysalo@fgi.fi; anssi.krooks@fgi.fi; paula.litkey@fgi.fi

**Keywords:** Intensity, laser scanning, natural samples, radiometric calibration, reflectance

## Abstract

We have studied the possibility of calibrating airborne laser scanning (ALS) intensity data, using land targets typically available in urban areas. For this purpose, a test area around Espoonlahti Harbor, Espoo, Finland, for which a long time series of ALS campaigns is available, was selected. Different target samples (beach sand, concrete, asphalt, different types of gravel) were collected and measured in the laboratory. Using tarps, which have certain backscattering properties, the natural samples were calibrated and studied, taking into account the atmospheric effect, incidence angle and flying height. Using data from different flights and altitudes, a time series for the natural samples was generated. Studying the stability of the samples, we could obtain information on the most ideal types of natural targets for ALS radiometric calibration. Using the selected natural samples as reference, the ALS points of typical land targets were calibrated again and examined. Results showed the need for more accurate ground reference data, before using natural samples in ALS intensity data calibration. Also, the NIR camera-based field system was used for collecting ground reference data. This system proved to be a good means for collecting *in situ* reference data, especially for targets with inhomogeneous surface reflection properties.

## 1. Introduction

There is a wide range of applications for airborne laser scanning (ALS) data. It can be used to monitor changes in, e.g., forests, built areas, or remote areas such as glaciers, creating 3D models, mapping shallow waters (bathymetry) etc. [1,2]. The result of a typical ALS survey is a point cloud, where every point has an X, Y and Z coordinate, which is determined with the help of GPS (*Global Positioning System*) and IMU (*Inertial Measurement Unit*). Additionally, the intensity value for each point is recorded. The intensity values are recorded in digital numbers (DN) and represent the laser returns, which are proportional to the number of photons impinging on the detector [3]. In physical terms it means the power entering the receiver. From the radar equation, the power that is detected by the receiver can be written as [4]:

(1) $$P_{r}=\frac{P_{t}D_{r}^{2}}{{4\pi R}^{4}\beta_{t}^{2}}\sigma$$

where *P_t_* is the transmitted power, *D_r_* is the receiver aperture, *R* is the range, *β_t_* is the transmitted beam width, and *σ* is the cross-section. The received power is dependent on the physical properties of the sensor, the distance between the sensor and the object, backscattering properties of the object, and the transmitted power. The latter one is problematic, because the transmitted power is usually unknown. The transmitted power is related to the pulse repetition frequency (PRF). The higher the PRF value, the lower the pulse energy is. The pulse energies for certain PRF values have been published for Optech systems [1] and those values have been used in calibration procedures [5, 6]. Reflectance is mostly defined as the total fraction of the incident (collimated) power on the unit surface area scattered into upper hemisphere by unit area of surface. Laser scanner measure only the fraction of reflectance that is retroreflected into the direction of illumination (0° angle between light source and detector), which we here call the backscattered reflectance.

The Finnish Geodetic Institute (FGI) has developed an empirical calibration scheme of ALS intensity data with portable brightness targets, such as tarps or gravel [6,7]. The tarps were measured in the laboratory [8] to get the exact backscattering properties, and laboratory results were compared to terrestrial laser scanner (TLS) and ALS data [9]. Commercially available brightness targets were also used to calibrate ALS data [10]. Those studies showed that the tarps and some commercially available targets can be used to calibrate intensity data. The aim of this paper is to present the possibility to use natural brightness targets for ALS intensity data calibration, since it is not always possible to use tarps or commercial gravel. This would also enable the intensity calibration for flights where any reference targets have not been used. The applications for corrected ALS intensity data can be found in [11,12].

In Section 2 the study area and the flight campaigns are described. The ALS intensity data correction is described in Section 3. Section 4 describes the results of the comparison of different natural targets and also NIR camera results. The conclusions and problems are discussed in Section 5.

## 2. Study area and data

### 2.1. Study area and airborne laser scanning campaigns

**Figure 1.**
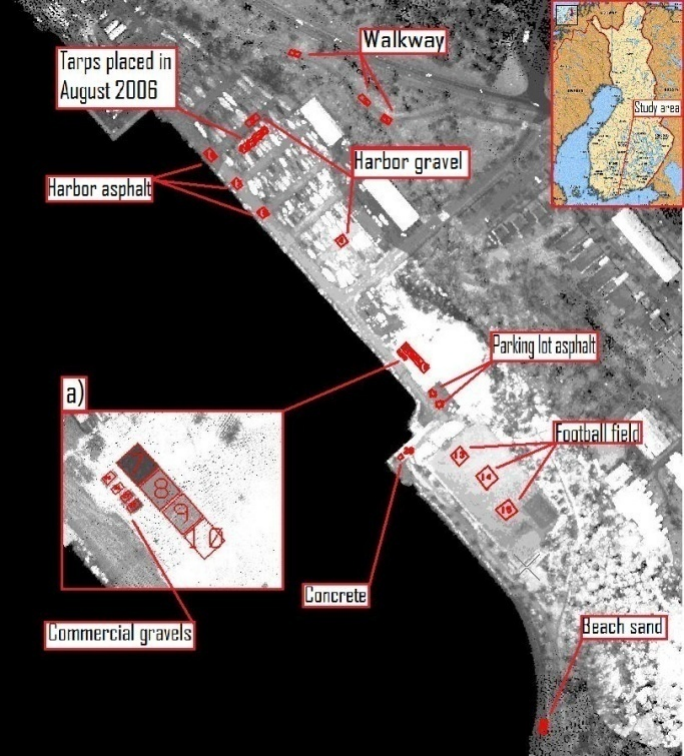
Lidar raw intensity image from a December 2006 flight. Placement of natural and commercial samples near Espoonlahti Harbor. (a) Tarps and commercial gravel during the December 2006 flights. Tarps from top to bottom: 5%, 25%, 30% and 45% nominal reflectance. Commercial gravel from top to bottom: Sanding Gravel, Yellow Quartz, Black Diabase and LECA.

The study area is situated in Espoonlahti Harbor, near Helsinki in South Finland (see Figure 1). The area has been an object of numerous airborne and terrestrial laser scanning campaigns and development of methods (e.g., creating 3D models of built areas).

There have been five ALS campaigns since 2004 with four different sensors, from altitudes 110 m to 2,200 m AGL (*Above Ground Level*). The first campaign took place on 29^th^ June 2004, and the sensor used was Optech ALTM 2033. In July 2005, the Optech ALTM 3100 scanner was used, in August and December 2006 Topeye MK-II, and in April 2007 Leica ALS50-II scanner. Detailed information about the flights and scanners is given in Table 1.

### 2.2. Samples and reference data

**Figure 2.**
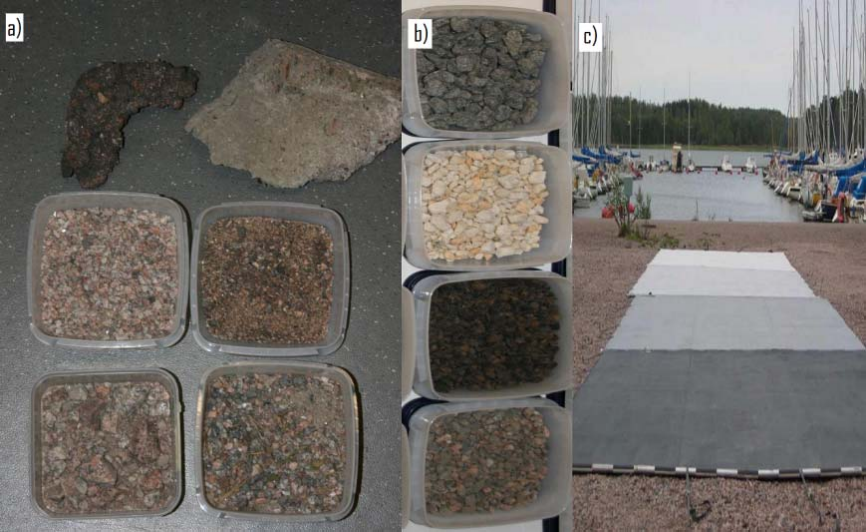
(a) Natural samples collected in Espoonlahti Harbor, from top left: asphalt, concrete, football field gravel, beach sand, harbor gravel and walkway gravel. (b) Commercial gravel that was used during the December 2006 flight. From top to bottom: Diabase, Quartz, LECA and Gravel. (c) Brightness tarps on the ground during the August 2006 flight: 10%, 30%, 50% and 70% nominal reflectance.

The sample data were collected near the Espoonlahti Harbor. Figure 2 shows the collected samples, commercial gravel and tarps that were laid down during the flights of August 2006. To obtain a wider spectrum of samples, concrete and asphalt samples were also included. Asphalt samples were collected from the parking lot and harbor road (see Figure 1, where the harbor road asphalt is brighter than the parking lot asphalt), a concrete sample, different gravel samples from a football field, walkway and harbor and a sand sample from the beach were also collected. The collected samples were measured in the laboratory to get the exact backscattering properties. A 1,064 nm Nd:YAG laser and CCD camera were used for the measurements. The set-up and the measurements technique are explained thoroughly in [8,9]. The 1064 nm wavelength is the same that the ALS systems use.

Commercially available gravel was used during the campaigns in December 2006 and April 2007. The gravel samples from the 2007 flights were too small to see in the laser data (the sizes of the gravel samples were too small to get enough laser returns to use the gravel as reference in calibration procedure). In 2006, the commercial gravel samples used were: black diabase (Diabase), yellow quartz (Quartz), Light Expanded Clay Aggregate, which consists of lightweight particles of burnt clay (LECA) and coarse gravel used for sanding the roads (Gravel). Tests have showed that these types of gravel can be used in ALS intensity calibration procedure [10].

Brightness tarps were used during the campaigns in August and December 2006. Targets of 10%, 30%, 50% and 70% nominal reflectance were used in August and targets of 5%, 25%, 30% and 45% nominal reflectance in December. More information about the brightness reference targets can be found in [8]. The brightness tarps are used in this study as validation targets, i.e., to calibrate laser points of natural brightness targets (e.g., sand and gravel). Knowing the exact backscattering properties for those tarps, other samples can be corrected [6].

**Figure 3.**
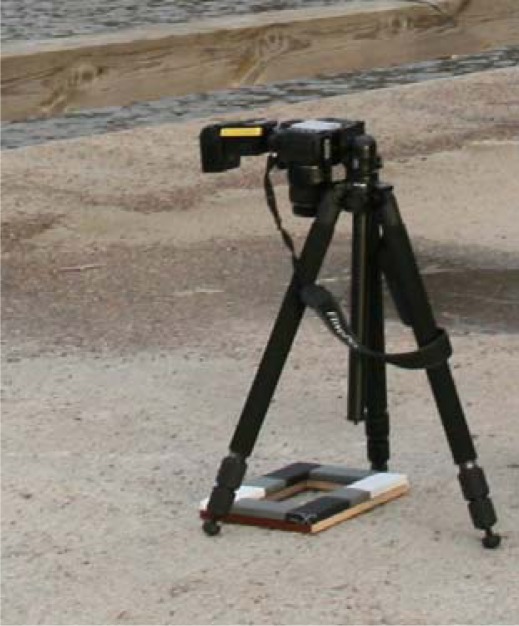
Measurements of concrete in Kivenlahti Harbor with Fuji IS PRO camera and the calibration frame.

To get a sample of a natural target for laboratory measurement is not always an easy task (e.g. in case of asphalt or concrete). Because of this, we developed an NIR camera-based field system for reference measurements. A Fuji IS PRO with an 850 nm IR-filter and ISO 100 1/250s exposure time was used with a Nikon SB800 flash, for which the output power variation was about 2%. A calibration frame (295 × 210 mm) was placed around the target to measure the reflectance (see Figure. 3). The frame cover is made of commercial white balance and exposure calibration target Lastolite XpoBalance, which has linear spectral response from 400 to 1,000 nm. To avoid shelf shadowing effect, only these areas of the target are selected, that have no shadows.

This system allows us to take reflectance measurements, without collecting samples and measuring them in the laboratory. The NIR camera is useful for collecting the *in situ* reference data. The NIR camera application gives the larger bulk of data for the area of interest than spectrometers, which gives us an opportunity to understand more about the reflectance variations within one sample (e.g. beach sand, for which the surface brightness showed some spatial variation).

## 3. Airborne laser scanner intensity data correction

**Figure 4.**
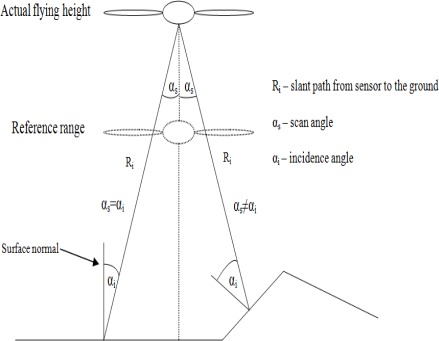
Difference between incidence angle and scan angle.

The laser points for each sample area were extracted, using the TerraScan (Terrasolid Ltd) program. The sample areas were chosen so, that they would be on a plane surface. This allows us to approximate the scan angle to be the same as the incidence angle and makes computation easier. The incidence angle is defined as an angle between surface normal and incoming laser beam. In the case of flat surfaces, the scan angle and incidence angle coincide (see Figure 4).

We assume the surfaces to have Lambertian backscatter properties. The incidence or scan angle effect in our case causes the reduction in the amount of light coming back to the sensor and could be corrected by multiplying the intensity value with 1/cosα [5], where α is the incidence angle. The incidence angle for each point can be calculated from the coordinates of the laser point and the scanner position.

In this study, there are several flights with different altitudes. The flying height plays an important role to the received power, which is related to the intensity. The inverse range-square dependency on the intensity value is called spherical loss [5,6]. The higher the flying altitude, the lower is the received power. If there are flights with multiple flying heights, the reference range should be selected (see Figure. 4). For example, if there are flights at 1,000 m, 200 m and 500 m altitudes, then we choose one of the heights as a reference range (e.g., 200 m). By multiplying the raw intensity values by the range squared, divided by the reference range squared (see Figure. 4), the effect of the energy loss due to the flying height is compensated and the intensity values from different heights are comparable.

Because the laser beam is travelling through the atmosphere, it is affected by the components and the conditions of the atmosphere. This is called the atmospheric effect. The exact atmospheric conditions are very difficult to obtain. Therefore, a MODTRAN Ver. 3 program for modeling the atmospheric conditions is used. This program calculates the total atmospheric transmittance, using the program’s inner atmospheric layers and user-defined input parameters. In this study, a mid-latitude summer model and visibility of 23 km (Espoonlahti Harbor is situated in a suburban area) was used. Other input parameters were: flying height, path length (assumed here to be the same as flying heights), and the wavelength range. Since all the sensors use 1,064 nm wavelength, the wavelength range was chosen from 1,063 nm to 1,065 nm. The path length is the distance over what the program calculates the total transmittance. The raw intensity values for atmospheric effect can be corrected by multiplying with 1/T^2^ [6] (because the laser beam travels from sensor to the ground and back), where T is the total transmittance calculated by MODTRAN Ver. 3.

The amount of energy that the laser uses is connected to the pulse repetition frequency (PRF). With high PRF values, the amount of energy that is transmitted with every pulse is lower than with the low PRF values [5,13]. The other important factor is pulse width. It is usually a few nanoseconds and is defined to be the time when the pulse power is continuously above half its maximum [13]. Pulses with shorter pulse width have higher peak power and higher pulse energy.

The calibration for different pulse energies is discussed in [5,6]. The main principle is to choose a reference PRF setting with defined pulse energy value, and divide it with the pulse energy value of the PRF setting in the current flight. This means that the correction value for pulse energy losses is a ratio between the reference pulse energy value and a pulse energy value in the current flightline. But the usual problem with the ALS data is that the relation between pulse energy and PRF is not known. Therefore, it is difficult to calibrate for the pulse energy losses. In our study, it does not have a crucial effect on the results, since we are looking for the stability of the targets within one type of sensor rather than between different sensors. The backscattered reflectance values for natural targets in this study are calculated, using reference targets from the same flying height. This cancels out the PRF correction, because it was the same for both, the natural target and the reference target. The pulse energy values for Optech scanners for certain PRF settings are reported in [1].

Summarizing the previous text, we have corrected the raw intensity values with the incidence angle correction, range correction and the atmospheric correction, leaving out the pulse energy correction. The equation for ALS intensity data correction can be written as:

(2) $$I_{\mathrm{corrected}}=I_{\mathrm{original}}\cdot \frac{R_{i}^{2}}{R_{\mathrm{ref}}^{2}}\cdot \frac{1}{\text{cos}\;\alpha }\cdot \frac{1}{T^{2}}\cdot \frac{E_{\mathrm{Tref}}}{E_{Tj}},$$

where I_original_ is the raw intensity value, R_i_ is the slope distance (see Figure. 4) from the sensor to the ground, R_ref_ is the chosen reference distance, α is the incidence angle (because we use samples from flat areas, the incidence angle is the same as the scan angle), T is the total atmospheric transmittance, E_Tref_ is the chosen reference pulse energy value and E_Tj_ is the pulse energy value in a current flightline.

**Figure 5.**
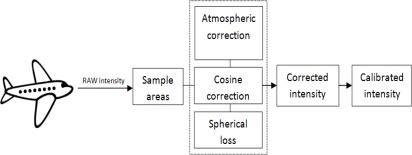
General workflow of calibrating the airborne laser scanner intensity data.

General workflow of calculating calibrated intensity values is shown in Figure. 5. The sample areas are selected from the raw laser data from the airborne survey. The extracted laser points are corrected with the incidence angel correction, range correction and atmospheric correction. The corrected intensity values are calibrated with the reference target by dividing the corrected intensity value of the natural target by the corrected intensity value of the calibration target and multiplying that ratio with the reflectance value of the calibration target that was measured in the laboratory.

## 4. Results

### 4.1. Stability study of natural brightness targets

For each sample area, laser points were corrected individually according to Eq. 2. Using the corrected intensity values, average intensity was calculated for each sample area. The number of points collected for each sample is summarized in Table 2. There is a long data series for all the natural samples, which allows us to compare laser data from different sensors and measurement conditions. Since there were variations in reflectance levels between different sensors, the stability of that target was investigated within the reflectance data obtained with a single sensor.

Using the data from flights where the tarps were used, the reflectance values for natural samples were calculated. Natural samples from the December 2006 and August 2006 campaigns were calibrated according to the 45 % tarp and the 30% tarp, respectively. The results are shown in Figure 6. For some natural targets, several samples were collected from the same flying height (see Figure 1). For example, for walkway, three samples were collected at the flying height of 110 m. All those samples are plotted in Figure 6.

The stability of natural brightness targets can be studied from Figure 6. The standard deviation of the walkway is noticeably large. There is also large deviation between the reflectance values. This indicates that the walkway is not stable (i.e., there is great deviation in the reflectance values) and is not suitable as a reference target. The large deviation may be caused by the fact that the walkway is surrounded by trees, which have an effect on the intensity values, but this also indicates that the sample is not homogeneous (see also [5]). It is also noticeable that the backscatter reflectance values from August flight in 2006 at the height of 320 m are higher (for all targets except for the harbor asphalt and parking lot asphalt). This could be explained by the fact that the moisture level in natural samples may have been higher in December than in August. The higher moisture level in samples decreases the backscatter reflectance values. This shows the need for more field measurements during the campaign. Here, the NIR camera system comes into use. Though the NIR camera works with different measurement geometry than lasers, it provides *in situ* results, which are closer to the actual conditions of the flight than the laboratory measurements. We have used backscatter reflectance values from the natural targets that have been collected since 2006 and measured in the laboratory with 1,064 nm Nd:YAG laser (as described in Sect. 2.1). If there is a change in the moisture conditions of the target collected for laboratory measurements, this may cause some deviation between the laboratory and flight data. Therefore, field measurements (especially when carried out simultaneously with the ALS flight) may provide a more reliable reference in such cases.

From the results in Figure 5, the harbor asphalt and parking lot asphalt seem to be the most stable targets. The football field, concrete and beach sand have also produced somewhat stable results. The walkway and harbor gravel (rough surface) deviate in large scale and are not reliable as calibration targets.

### 4.2. Using natural targets in reflectance calibration

To test the use of different natural samples as reference targets, backscatter reflectances for all other natural samples were calculated using each of these samples as a calibration target. The results are shown in Figure 7. The type of sensor and altitudes is plotted on the x-axis, and the calibrated backscatter reflectance is on the y-axis. There is a separate plot for every natural sample with the names of the reference targets in the legend. For example, the concrete sample (Figure 7G) is calibrated using the parking lot and harbor asphalt, football field, beach sand, and 30% tarp intensity values (obtained from the laboratory measurements) to get the backscatter reflectance for concrete. The NIR camera reflectance value was used for parking lot asphalt, because there was no laboratory sample collected from this target.

The backscatter reflectance values from Optech 2004 (Optech_2033) and 2005 (Optech_3100) are considerably different from those obtained from other campaigns and sensors (Figure. 7). Since our study of laser scanner intensity using ground targets only started with the following campaigns, no ground reference is available from the Optech campaigns, and the calibration values are taken from the samples measured during the later campaigns. This may explain some of the discrepancy with the other results, and especially points out the importance of obtaining the ground reference simultaneously with the flight. Differences between Topeye MK-II and Leica ALS50-II can also be noticed. The deviation with the Leica sensor can be explained by AGC (*Automatic Gain Control*), which changes the intensity values (see also [10]). A further study about the effect of AGC on intensity values is underway.

The walkway and harbor gravel do not show reliable results, even using the 30% reflectance tarp as a reference target. The results deviate in large scale. This is likely to be caused, as mentioned above, by the fact that the walkway is surrounded by trees and the harbor gravel has a rough surface and large scale inhomogeneity in surface properties [see Figure 2 (a)]. The backscatter reflectance values for parking lot and beach sand with different natural samples as reference targets vary in small scale between Topeye and Leica sensors. This indicates that these targets are stable enough to use them as reference. They have also produced consistent reflectance values with the 30% tarp when used as reference for other natural samples (see, e.g. Figure. 7F, where the reflectance values for harbor asphalt calibrated with the parking lot asphalt, beach sand, and the 30% tarp are closest to each other).

To present an example of using a single target in the calibration, the results of using parking lot asphalt as reference to the other natural samples is shown in Figure. 8. We have left out the results from the 2004 and 2005 Optech sensor, because no reference measurements were available at that time.

We have compared the similar results from laboratory measurements, also plotted in Figure 8, using the parking lot asphalt as reference. The targets with the best stability over all (or most) of the measurements (such as harbor asphalt and football field) also show a similarity in the laboratory and NIR camera results. However, there are discrepancies between different sensors for these targets as well. Whether this is an effect of sensor parameters or different measurement conditions (such as weather) are an object of a further study. Even though the backscatter reflectance values of the walkway (Topeye December and Leica sensor data) are similar to the laboratory results, the standard deviation of walkway data is too large to consider it a stable target.

### 4.3. Digital camera results

**Figure 9.**
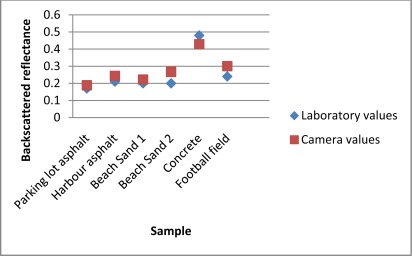
NIR camera results compared to the laser backscatter values of the field targets.

The reflectance’s of all the field targets measured with the Fuji IS-PRO digital camera are presented and compared with the laboratory laser backscatter measurements (of the laboratory samples of these targets) in Figure. 9.

The reflectance levels are consistent for the samples that showed stability over a time span of ALS measurements (see Sect. 4.1), such as the asphalts, whereas those with larger variation in ALS and laboratory laser results (e.g., concrete) have also produced more deviation between NIR camera and laser reflectance’s. However, some of the differences may be caused by the differences in measurement geometry between the digital camera and the laboratory backscatter measurement, but overall, the reflectance levels are repeated for almost all samples.

The comparison also demonstrates the need of using an *in situ* means for reference measurement (such as the camera approach in this study). Comparing the beach sand NIR camera results with laboratory measurements showed a 7% variation in backscattered reflectance values. This may be due to the fact that the NIR camera results represent a larger area of the target than the small laboratory sample. Even though the laboratory backscatter instrument is better capable of reproducing the ALS backscatter geometry, camera images can be collected from a larger area of the target than just a small laboratory sample. This is an advantage for targets with inhomogeneity in surface reflectance, such as the beach sand, for which the NIR camera images and laboratory samples acquired from different spots have produced slightly different reflectances.

## 5. Conclusions

We have investigated the use of natural targets as references in ALS intensity calibration. We have found that the stability of the target plays a crucial role, e.g., the asphalts showed better stability over different campaigns than beach sand or harbor gravel investigated in this study. Also, getting reliable ground reference (reflectance) values is a challenge, which requires more accurate measurements on the field. We have shown that the NIR camera system offers a good possibility to get *in situ* reference data, even for targets with inhomogeneity in surface reflection properties.

Some discrepancy exists between different sensors, which require further studies. The transmitted power is usually unknown. Different sensors have different power settings, which causes variation in intensity values. There is also a question of the automatic gain control (AGC), such as that in the Leica ALS 50-II systems. Since it changes the intensity values, it is important to get to know how, and on what scale, it changes the values.

The changes in reference targets over time also cause deviations between campaigns. Repeated time series for different samples should be studied to understand the changes in reference targets. Weather conditions affect the results, especially surface moisture, which should be monitored with ground instruments.

This is one of the first studies in the field of ALS, where intensity data are studied over a longer time span. A limited amount of data with ground reference are thus far available for the same location, collected with different instruments and at varying conditions, to study the effects of different parameters on the intensity calibration. Nevertheless, the absolute radiometric calibration (e.g., using a reference target as in this study) should be independent on the sensor used. Therefore, a large bulk of data from different (repeated) experiments is still needed to know on a more general scale, which type of (natural) targets are suitable for ALS radiometric calibration and the requirements of the calibration procedure itself, to produce meaningful results in the future ALS campaigns.

## Figures and Tables

**Figure 6A:**
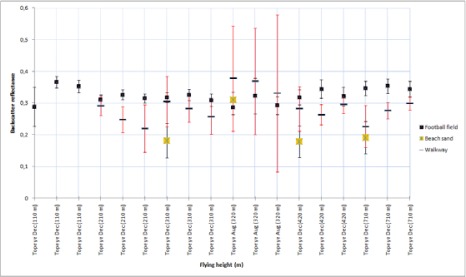
Backscatter reflectance values calibrated with 45% tarp. The intensity values at 320 m flight altitude are relative to 30% tarp. The standard deviation for the walkway is marked with red color as noticeably large. Data points from heights 110 m, 210 m, 310 m, 460 m and 710 m are from the December 2006 campaign, and data points from height 320 m are from August 2006 campaign. Results for football field, beach sand and walkway.

**Figure 6B:**
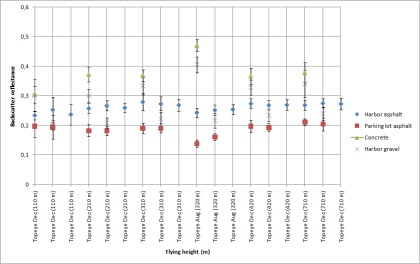
Backscatter reflectance values calibrated with 45% tarp. The intensity values at 320 m flight altitude are relative to 30% tarp. Data points from heights 110 m, 210 m, 310 m, 460 m and 710 m are from the December 2006 campaign, and data points from height 320 m are from August 2006 campaign. Results for parking lot asphalt, harbor asphalt, harbor gravel and concrete.

**Figure. 7A.**
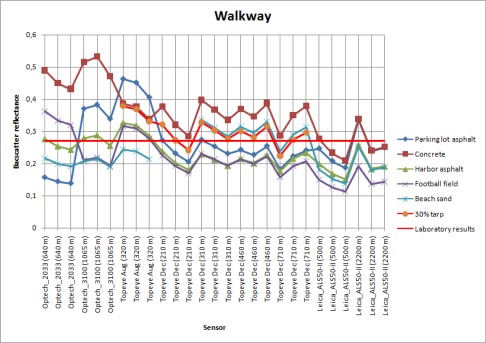
Backscatter reflectances of the walkway sample calibrated with different samples as reference.

**Figure. 7B.**
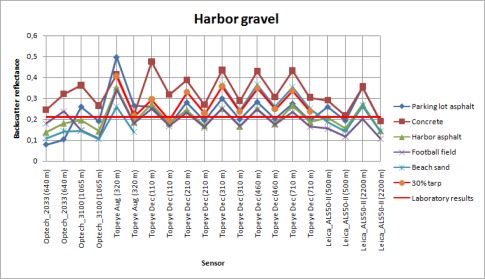
Backscatter reflectances of the harbor gravel sample calibrated with different samples as reference.

**Figure. 7C.**
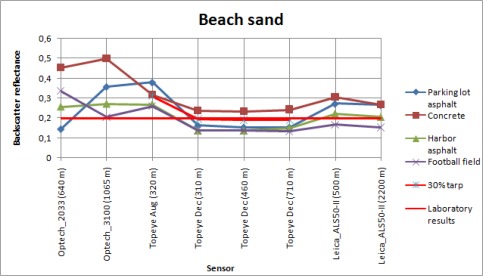
Backscatter reflectances of the beach sand sample calibrated with different samples as reference.

**Figure. 7D.**
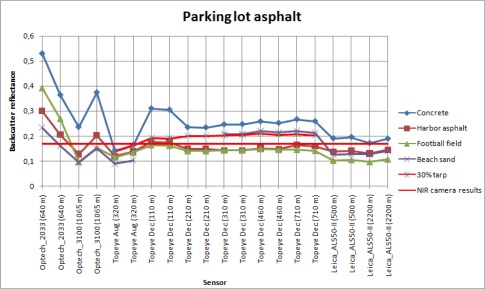
Backscatter reflectances of the parking lot asphalt sample calibrated with different samples as reference.

**Figure. 7E.**
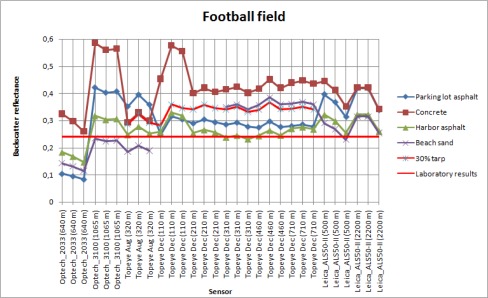
Backscatter reflectances of the football field sample calibrated with different samples as reference.

**Figure. 7F.**
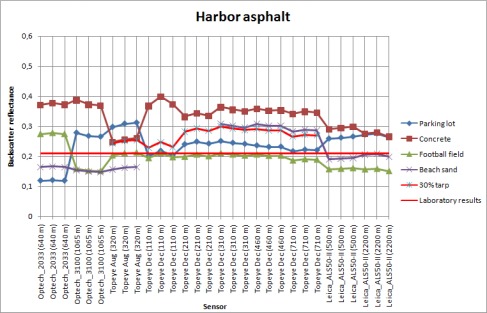
Backscatter reflectances of the harbor asphalt sample calibrated with different samples as reference.

**Figure. 7G.**
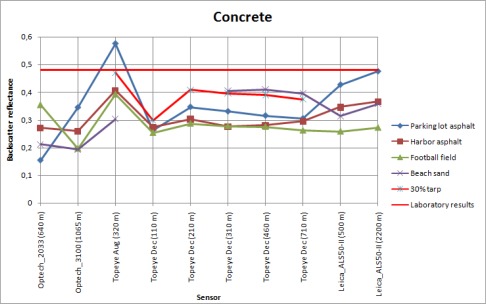
Backscatter reflectances of the concrete sample calibrated with different samples as reference.

**Figure 8A:**
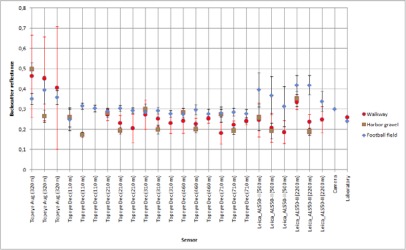
Backscatter reflectance values of natural samples using parking lot asphalt as reference target. Standard deviation of the walkway is marked with red as the largest one. Backscatter reflectance for walkway, harbor gravel and football field.

**Figure 8B:**
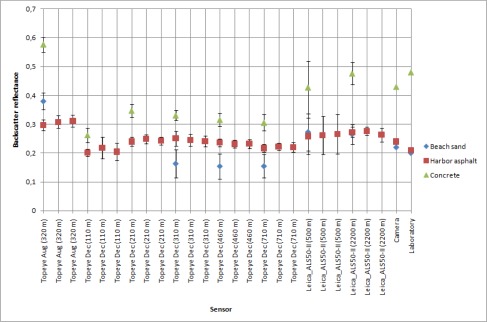
Backscatter reflectance values of natural samples using parking lot asphalt as reference target. Standard deviation of the walkway is marked with red as the largest one. Backscatter reflectance for beach sand, harbor asphalt and concrete.

**Table 1.** Flight campaigns in Espoonlahti Harbor. Date when the campaign took place, scanner used, flying altitudes, average point density and laser footprint size on the ground.

**Date**	**Scanner**	**Wavelength, nm**	**Flying altitude (AGL), m**	**Average point density, pts/m^2^**	**Laser footprint size on the ground, m**
Jun. 29, 2004	Optech ALTM 2033	1064	640	9.2	0.13
Jul. 12, 2005	Optech ALTM 3100	1064	1065	7.3	0.32
Aug. 31, 2006	Topeye MK-II	1064	320	13.7	0.32
Dec. 18, 2006	Topeye MK-II	1064	110; 210; 310; 460; 710	63.5; 17.5; 16.3; 6.6; 3.5	0.11; 0.21; 0.31; 0.46; 0.71
Apr. 26, 2007	Leica ALS50-II	1064	500; 2200	5.5; 0.5	0.11; 0.48

**Table 2.** Data series of samples taken from the ALS data. Number of points collected for every sample and flying height.

**Sample**	**Flying altitude AGL, m** **Number of points collected**									
	**Topeye MK-II December**			**Topeye MK-II August**	**Topeye MK-II December**	**Leica ALS50-II**	**Optech ALTM 2033**	**Topeye MK-II December**	**Optech ALTM 3100**	**Leica ALS50-II**
	110	210	310	320	460	500	640	710	1065	2200
Walkway	-	543	1224	751	455	871	462	143	191	44
Harbour gravel	4535	785	1345	2236	488	600	484	87	273	47
Beach sand	-	-	172	856	42	332	228	54	63	23
Parking lot asphalt	1703	577	450	450	101	192	257	14	108	10
Football field	14424	5623	5294	4488	1747	2396	2272	787	1133	165
Harbour asphalt	3656	1409	1195	541	573	802	659	199	398	66
Concrete	489	64	195	34	19	51	44	4	6	4
Tarp 5%	1612	514	229	-	109	-	-	79	-	-
Tarp 8%	-	-	-	945	-	-	-	-	-	-
Tarp 20%	1426	510	240	-	48	-	-	53	-	-
Tarp 26%	1263	594	762	273	48	-	-	225	-	-
Tarp 40%	2816	443	671	-	53	-	-	121	-	-
Tarp 50%	-	-	-	297	-	-	-	-	-	-
Tarp 70%	-	-	-	234	-	-	-	-	-	-
Gravel	183	54	30	-	9	-	-	31	-	-
Quartz	138	60	23	-	5	-	-	26	-	-
Diabase	133	48	19	-	5	-	-	2	-	-
LECA	136	50	26	-	6	-	-	7	-	-
